# Reply to: Genetic differentiation at probe SNPs leads to spurious results in meQTL discovery

**DOI:** 10.1038/s42003-023-05646-9

**Published:** 2023-12-21

**Authors:** Youshu Cheng, Boyang Li, Xinyu Zhang, Bradley E. Aouizerat, Hongyu Zhao, Ke Xu

**Affiliations:** 1 Department of Biostatistics, School of Public Health, Yale University, New Haven, CT USA; 2 VA Connecticut Healthcare System, US Department of Veterans Affairs, West Haven, CT USA; 3 Department of Psychiatry, Yale School of Medicine, New Haven, CT USA; 4 Bluestone Center for Clinical Research, New York University, New York, NY USA; 5 Department of Oral and Maxillofacial Surgery, New York University, New York, NY USA

**Keywords:** DNA methylation, Epigenomics

**replying to** G.L. Meeks et al. *Communications Biology* 10.1038/s42003-023-05658-5 (2023).

In the paper by Li et al.^1^, we reported DNA methylation (DNAm) of 946 CpG sites influenced by genetic variants in the conventional model (LA-naïve), and 135 CpGs with genetic effects that significantly differed by local ancestry (LA-specific). Meeks et al. raised a concern that 37.5% of the 946 CpG sites were inadequately controlled as they contained nearby commons variants (Probe-SNPs). They argued that different allele frequencies of probe-SNP between populations of African ancestry and European ancestry led to spurious findings in the paper. In response to Meeks et al.’s concern, using a Methyl-Seq approach in a subset of the samples (N = 211), we were able to show that a large proportion (47.1%-71.6%) of the significant SNP-CpG associations originally reported by Li et al. using Illumina HumanMethylation 450K (HM450K)^1^ were replicated. We did not observe significant difference in the replication rates between CpGs with and without probe-SNPs, indicating that there is no substantial evidence to suggest that CpGs with probe-SNPs biased our published findings.

## Associations of the reported SNP-CpG pairs are replicated using a bisulfite sequencing method

It is well known that the methylation probe containing SNP within 10 base pair (bp) biases the call of CpG methylation in an array-based assay^2,3^. In practice, CpG sites with SNP within 10bp are filtered out in analyses^4^ although a few studies removed such CpG sites beyond 10bp window^5^. Other studies examined the effects of probe-SNP on significant CpG sites in post-hoc analyses^6^. In our paper^1^, we removed the polymorphic CpG sites (the ones that overlay with SNPs) and CpG sites with probe-SNP within 10bp based on the annotation file provided by Illumina Infinium. Additionally, following the previous report^7^, we removed CpG sites with detection *p*-value > 1e−12, a more stringent threshold than recommended by Illumina (*p* = 0.01). The use of a stringent detection p-value could more effectively filter out low quality CpG sites and enhance the quality of DNAm array data^8^.

**Fig. 1:**
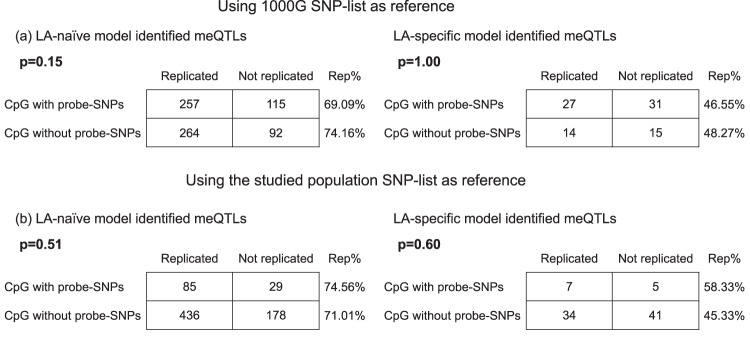
Comparing the replication rate between CpGs with probe-SNPs and CpGs without probe-SNPs. The Methyl-seq data (*N* = 211) were used to replicate the meQTLs identified by Li et al. CpG sites with common probe-SNP within 50bp were defined using the SNPs in **a** the 1000 Genomes and **b** the study sample population, respectively. The *p*-values were derived from *χ*^2^ test.

Methyl-seq serves as a gold standard to validate array-based methylation detection^9,10^. To confirm the quality of DNA methylation in our previous study, we re-profiled DNA methylation of 211 samples (Supplementary Table 1) that were included in the previous paper^1^ using Agilent SureSelectXT Methyl-Seq (Supplementary Data 1). No significant differences in demographic variables were observed between the 211 samples with Methyl-seq data and the original discovery group except for smoking (*p* = 0.04) (Supplementary Table 1). A total of 547 out of the 946 CpG sites in the conventional model, and 77 out of 135 CpGs in the LA-specific model were measured by both platforms. The 547 CpGs in the conventional model had 728 significant SNP-CpG associations, while the 77 CpGs in the LA-specific model had 87 significant associations. To replicate the original findings identified using HM450K data, we first re-conducted the association analyses for the significant SNP-CpG pairs using Methyl-seq data, then we investigated whether the replication rates would differ between CpGs with and without probe-SNPs. The overall replication rates were 71.6% for the 728 significant SNP-CpG pairs in the conventional model (Supplementary Data 2), and 47.1% for the 87 significant pairs in the LA-specific model (Supplementary Data 2). A similar trend of replication rates was observed in the original results of Li et al.^1^: the replication rate in the LA-specific model was consistently lower than that in the conventional model. Importantly, we found no significant difference in replication rates between CpGs with and without probe-SNPs (*p* = 0.15 for CpGs in the LA-naïve model; *p* = 1.00 for CpGs in the LA-specific model) (Fig. 1a).

Meeks et al. define whether a CpG has probe SNPs within 50bp using the 1000 genomes data. Here, we re-examined if our reported CpGs harbored nearby SNPs using the genotype data from the study cohort (4.7 million SNPs with minor allele frequency >0.01)^1^: among the 946 CpGs identified in the conventional model (LA-naïve), 28 (3.0%) had probe SNPs within 10bp and 157 (16.6%) had probe SNPs within 50bp. Among the 135 CpGs with genetic effects significantly differed by ancestry (LA-specific), only 3 of them (2.2%) had probe SNPs within 10bp and 21 of them (15.6%) had probe-SNPs within 50bp. When evaluating whether there is a significant difference in replication rates between CpGs with and without probe SNPs, we also presented parallel results using the studied population SNP-list as reference and found no significant difference (Fig. 1b). Together, these data suggest that there is no clear evidence for the probe-SNP bias in the results of Li et al.^1^ as the replication rate in CpGs with probe-SNPs was not significantly different from that in CpGs without probe-SNPs.

## The 1000 Genomes versus study population based genomes

Meeks et al. mapped the nearby SNPs for our reported CpG sites using the 1000 Genomes SNP-list and found a large proportion (37–61%) of them contained SNPs within 50bp. However, using the genotype data from our study cohort, we noted that the proportion of CpGs with probe SNPs was lower (2.2–3.0% containing SNPs within 10bp, 15.5–16.5% containing SNPs within 50bp) (Supplementary Fig 1). One reason for the discrepancy was the difference in size between the studied population SNP-list and the 1000 Genomes SNP-list, and we agreed with Meeks et al. that our strict genotype quality control steps^1^ led to the smaller SNP-list (4.7 million SNPs with minor allele frequency >0.01 in our studied cohort). This strict procedure is appropriate to keep high quality SNPs for meQTL identification, but to avoid probe-SNP bias, a more comprehensive SNP-list, such as the 1000 Genomes, is also a helpful reference to filter CpGs with probe-SNPs. Therefore, to assess whether our previous results were affected by potential probe-SNPs defined by 1000 Genomes, we performed parallel replication analyses using both the studied population SNP-list and the 1000 Genomes SNP-list as references, and the results were consistent: there was no significant difference in replication rates between CpGs with and without probe-SNPs (Fig. 1).

In summary, the concerns highlighted by Meeks et al. underscored the importance of filtering CpGs with probe-SNPs in methylation association and meQTL studies in a mixed ancestry population. Using Methyl-Seq data, we confirmed that replication rates of the significant SNP-CpG associations did not differ significantly between CpGs with and without probe-SNPs. Applying bisulfite methylation sequencing not only prevents the biased methylation detection influenced by nearby SNPs in array-based assay, but can also enable the evaluation of additional allelic/haplotypic genetic-epigenetic effects that array-based methods are blind to^11,12^. Furthermore, polymorphic repeats between human populations may require specialized long-read techniques^13^. Altogether, future studies may consider applying Methyl-seq based methods instead of relying on array-based assay.

## Methods

Quality control (QC) on the Methyl-seq data was conducted following standard procedure^14^. Quality of sequence data was examined by using FastQC (ver. 0.11.8). We used Bismark pipelines (ver. v0.22.1_dev)^15^ to align the reads to the bisulfite human genome (hg19) with default parameters. Quality-trimmed paired-end reads were transformed into a bisulfite converted forward strand version (C → T conversion) or into a bisulfite-treated reverse strand (G → A conversion of the forward strand). Duplicated reads were removed from the Bismark mapping output by *deduplicate_bismark*. All CpG sites were grouped by sequencing coverage, also known as read depth. Only the CpG sites with coverage > 10x depth were kept to ensure the data quality. The study was approved by the committee of the Human Research Subject Protection at Yale University and the Institutional Research Board Committee of the Connecticut Veteran Healthcare System. Informed consent was obtained from all human participants. All analyses were carried out in accordance with all relevant ethical regulations.

### Supplementary information

Supplementary Information

Description of Additional Supplementary Files

Supplementary Data 1

Supplementary Data 2

## Data Availability

The generation of the methyl-seq data was partially supported by NIH grants. The full dataset will be released based on the NIH data sharing plan and Veterans Aging Cohort Study policy. All relevant methyl-seq data for the samples and CpGs involved in this manuscript are available in Supplementary Data 1.

## References

[1] Li B (2022). Incorporating local ancestry improves identification of ancestry-associated methylation signatures and meQTLs in African Americans. Commun. Biol..

[2] Price ME (2013). Additional annotation enhances potential for biologically-relevant analysis of the Illumina Infinium HumanMethylation450 BeadChip array. Epigenetics Chromatin.

[3] Naeem H (2014). Reducing the risk of false discovery enabling identification of biologically significant genome-wide methylation status using the HumanMethylation450 array. BMC Genomics.

[4] Huan T (2019). Genome-wide identification of DNA methylation QTLs in whole blood highlights pathways for cardiovascular disease. Nat. Commun..

[5] Hannon E (2016). An integrated genetic-epigenetic analysis of schizophrenia: evidence for co-localization of genetic associations and differential DNA methylation. Genome Biol..

[6] Zhang L (2020). Epigenome-wide meta-analysis of DNA methylation differences in prefrontal cortex implicates the immune processes in Alzheimer’s disease. Nat. Commun..

[7] Lehne B (2015). A coherent approach for analysis of the Illumina HumanMethylation450 BeadChip improves data quality and performance in epigenome-wide association studies. Genome Biol..

[8] Heiss JA, Just AC (2019). Improved filtering of DNA methylation microarray data by detection p values and its impact on downstream analyses. Clin. Epigenetics.

[9] Kurdyukov S, Bullock M (2016). DNA methylation analysis: choosing the right method. Biology.

[10] Feng S, Zhong Z, Wang M, Jacobsen SE (2020). Efficient and accurate determination of genome-wide DNA methylation patterns in Arabidopsis thaliana with enzymatic methyl sequencing. Epigenetics Chromatin.

[11] Bell CG (2018). Obligatory and facilitative allelic variation in the DNA methylome within common disease-associated loci. Nat. Commun..

[12] Abante J, Fang Y, Feinberg AP, Goutsias J (2020). Detection of haplotype-dependent allele-specific DNA methylation in WGBS data. Nat. Commun..

[13] Sarkar A, Lanciano S, Cristofari G (2023). Targeted nanopore resequencing and methylation analysis of LINE-1 retrotransposons. Methods Mol. Biol..

[14] Wreczycka K (2017). Strategies for analyzing bisulfite sequencing data. J. Biotechnol..

[15] Krueger F, Andrews SR (2011). Bismark: a flexible aligner and methylation caller for Bisulfite-Seq applications. Bioinformatics.

