# Supplementary material for: Reply to: Genetic differentiation at probe SNPs leads to spurious results in meQTL discovery
Source: Commun Biol. 2023 Dec 21;6:1296. doi: 10.1038/s42003-023-05646-9 (PMC10739901; doi:10.1038/s42003-023-05646-9)
Supplement: Supplementary file 1 — Supplementary Information [file 42003_2023_5646_MOESM1_ESM.pdf]

Supplemental information

Reply to: **Genetic differentiation at probe SNPs leads to spurious results in meQTL discovery**

Youshu Cheng[1,2], Boyang Li[1,2], Xinyu Zhang[2,3], Bradley E. Aouizerat[4,5], Hongyu Zhao[1,2] & Ke Xu[2,3]

1. Department of Biostatistics, School of Public Health, Yale University, New Haven, CT, United States.
2. VA Connecticut Healthcare System, US Department of Veterans Affairs, West Haven, CT, United States.
3. Department of Psychiatry, Yale School of Medicine, New Haven, CT, United States.
4. Bluestone Center for Clinical Research, New York University, New York, NY, United States.
5. Department of Oral and Maxillofacial Surgery, New York University, New York, NY, United States.

Corresponding to Ke Xu (ke.xu@yale.edu) and Hongyu Zhao (hongyu.zhao@yale.edu)

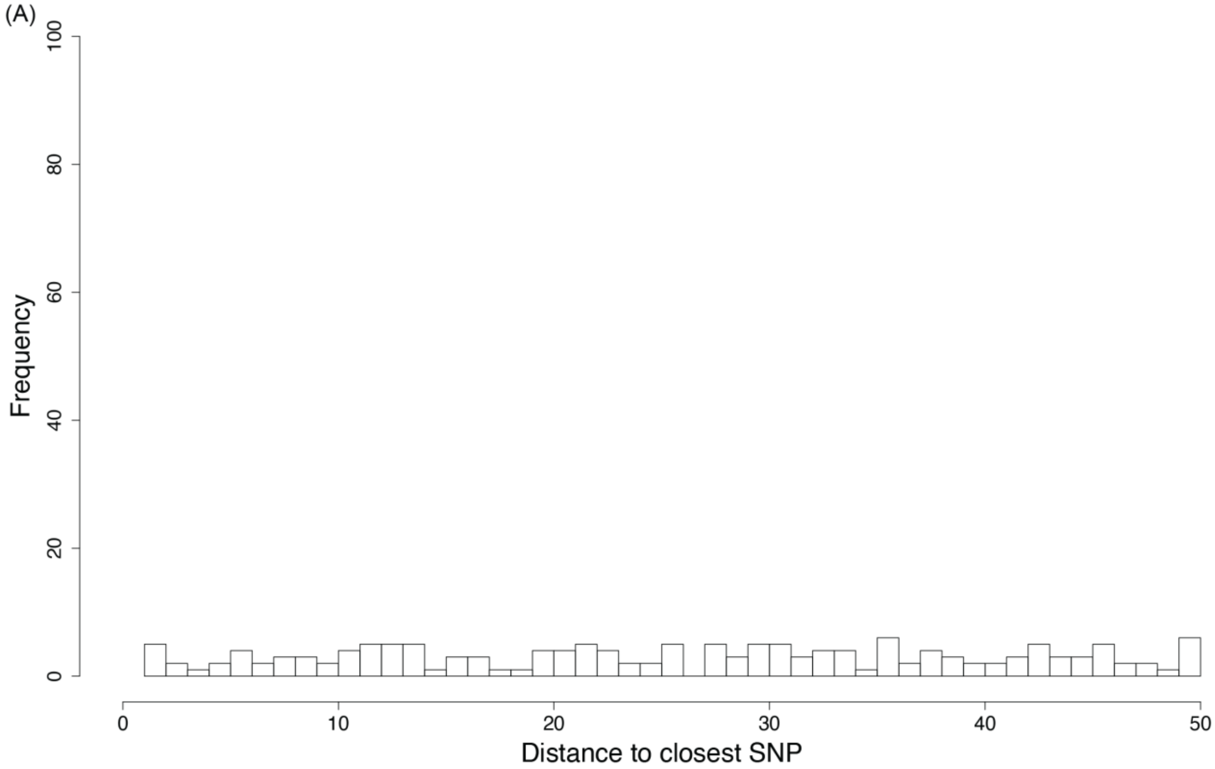

Supplementary Fig 1: Histogram showing the frequency of the distance from CpG to the corresponding probe SNP using genotype data from the study cohort. The histogram cells on the x-axis are intervals of the form [a, b), i.e., the first cell include the left-hand endpoint (0), but not the right one (1).

	Studied samples with Methyl-seq (n = 211)	P-value for comparison with the original group*
Sex-male (%)	100%	N/A
HIV-positive (%)	100%	N/A
Age (years)	50.07 ± 6.13	0.43
Adherence to medication (%)	79.13%	1.00
Viral load (log10)	2.67 ± 1.18	0.60
Smoking-smokers (%)	67.14%	0.04
Peth (log10)	1.69 ± 2.49	0.70
Global ancestry (proportion of AFR)	0.8086 ± 0.0970	0.83

Supplementary Table 1: Demographic information for the 211 samples with Methyl-seq data. *P-values were reported for the comparison between the samples with Methyl-seq data and the original discovery group (demographic information has been published by Li et al[1]).

Supplementary References

1. Li, B. *et al.* Incorporating local ancestry improves identification of ancestry-associated methylation signatures and meQTLs in African Americans. *Communications Biology* **5**, 401 (2022).
