# Supplementary material for: Reply to: Genetic differentiation at probe SNPs leads to spurious results in meQTL discovery
Source: Commun Biol. 2023 Dec 21;6:1296. doi: 10.1038/s42003-023-05646-9 (PMC10739901; doi:10.1038/s42003-023-05646-9)
Supplement: Supplementary file 2 — Description of Additional Supplementary Files [file 42003_2023_5646_MOESM2_ESM.pdf]

Description of Additional Supplementary Files

File name: Supplementary Data 1
Description: The Methyl-seq data relevant to this study.

File name: Supplementary Data 2
Description: The replication results for the significant SNP-CpG pairs in the conventional model and LA-specific model, and the source data for the main figure.
